# Roles of Pannexin-1 Channels in Inflammatory Response through the TLRs/NF-Kappa B Signaling Pathway Following Experimental Subarachnoid Hemorrhage in Rats

**DOI:** 10.3389/fnmol.2017.00175

**Published:** 2017-06-06

**Authors:** Ling-Yun Wu, Zhen-Nan Ye, Chen-Hui Zhou, Chun-Xi Wang, Guang-Bin Xie, Xiang-Sheng Zhang, Yong-Yue Gao, Zi-Huan Zhang, Meng-Liang Zhou, Zong Zhuang, Jing-Peng Liu, Chun-Hua Hang, Ji-Xin Shi

**Affiliations:** ^1^Department of Neurosurgery, Jinling Hospital, School of Medicine, Nanjing UniversityNanjing, China; ^2^Department of Neurosurgery, The Second Affiliated Hospital of Guangzhou Medical UniversityGuangzhou, China; ^3^Department of Neurosurgery, Zhongdu HospitalBengbu, China; ^4^Department of Neurosurgery, Jinling Hospital, School of Medicine, Southern Medical UniversityNanjing, China

**Keywords:** subarachnoid hemorrhage, pannexin-1, TLR, NF-κB, early brain injury

## Abstract

**Background:** Accumulating evidence suggests that neuroinflammation plays a critical role in early brain injury after subarachnoid hemorrhage (SAH). Pannexin-1 channels, as a member of gap junction proteins located on the plasma membrane, releases ATP, ions, second messengers, neurotransmitters, and molecules up to 1 kD into the extracellular space, when activated. Previous studies identified that the opening of Pannexin-1 channels is essential for cellular migration, apoptosis and especially inflammation, but its effects on inflammatory response in SAH model have not been explored yet.

**Methods:** Adult male Sprague-Dawley rats were divided into six groups: sham group (*n* = 20), SAH group (*n* = 20), SAH + LV-Scramble-ShRNA group (*n* = 20), SAH + LV-ShRNA-Panx1 group (*n* = 20), SAH + LV-NC group (*n* = 20), and SAH + LV-Panx1-EGFP group (*n* = 20). The rat SAH model was induced by injection of 0.3 ml fresh arterial, non-heparinized blood into the prechiasmatic cistern in 20 s. In SAH + LV-ShRNA-Panx1 group and SAH + LV-Panx1-EGFP group, lentivirus was administered via intracerebroventricular injection (i.c.v.) at 72 h before the induction of SAH. The Quantitative real-time polymerase chain reaction, electrophoretic mobility shift assay, enzyme-linked immunosorbent assay, immunofluorescence staining, and western blotting were performed to explore the potential interactive mechanism between Pannexin-1 channels and TLR2/TLR4/NF-κB-mediated signaling pathway. Cognitive and memory changes were investigated by the Morris water maze test.

**Results:** Administration with LV-ShRNA-Panx1 markedly decreased the expression levels of TLR2/4/NF-κB pathway-related agents in the brain cortex and significantly ameliorated neurological cognitive and memory deficits in this SAH model. On the contrary, administration of LV-Panx1-EGFP elevated the expressions of TLR2/4/NF-κB pathway-related agents, which correlated with augmented neuronal apoptosis.

**Conclusion:** Pannexin-1 channels may contribute to inflammatory response and neurobehavioral dysfunction through the TLR2/TLR4/NF-κB-mediated pathway signaling after SAH, suggesting a potential role of Pannexin-1 channels could be a potential therapeutic target for the treatment of SAH.

## Introduction

Subarachnoid hemorrhage (SAH), especially aneurysmal SAH, accounting for 5% of all stroke types, is a catastrophic cerebrovascular disease with high morbidity and mortality throughout the world. Eighty-five percent of all cases of SAH are due to cerebral aneurysm rupture (Connolly et al., 2012). Complications of SAH, including rebleeding, hydrocephalus, cerebral vasospasm and delayed cerebral ischemia, could lead to poor clinical outcomes. Despite improvements in understanding of pathophysiology and the management of SAH, current therapeutic strategies are still unsatisfactory and clinical outcomes remain disappointing (Chen et al., 2014).

Basic and clinical researches showed that the presence of delayed vasospasm is not a necessary step for either delayed ischemic neurological deficits (DINDs) or poor neurological outcome (Cossu et al., 2014). Recent advances in SAH research suggest that it is essential to target other equally important pathological mechanisms initiated within minutes or hours after the occurrence of SAH. Currently, the early brain injury (EBI), which refers to the acute injury to the whole brain within the first 72 h after SAH, is considered to be one of the primary causes of mortality and poor outcomes in SAH patients (Vergouwen et al., 2011; Chen et al., 2015). However, following the rupture of intracranial aneurysm, various pathophysiological events occur during the EBI period, such as the raised intracranial pressure, decreased cerebral blood flow and perfusion pressure, blood brain barrier (BBB) disruption, brain swelling, brain edema, acute vasospasm, and impaired cerebral auto-regulation (Fujii et al., 2013). Meanwhile, many previous researches implicate neuroinflammation as a key mediator of injury expansion and behavioral deficits. Novel treatment options targeting these inflammatory responses have proved efficacious in alleviating EBI, reducing delayed ischemia and improving long-term cognitive function (Lucke-Wold et al., 2016). Furthermore, during the inflammatory response process after SAH, activation of TLRs-mediated pathway is reported to initiate the inflammatory cascades and consequently damage neurons and white matter (Ascenzi et al., 2005; Pradilla et al., 2010). In addition, the signaling cascades initiated by TLR2 and TLR4 show much overlap and cooperativity. Activation of these receptors results in the activation of NF-κB via the MyD88-/TRIF-dependent pathways, which induces the upregulation of leukocyte adhesion molecules and immunomodulatory cytokines, and increases the production of pro-inflammatory cytokines. As a consequence, the release of cytokines contributes to neutrophil activation, adhesion and recruitment, which further exacerbates the inflammatory injury (van Beijnum et al., 2008; Vilahur and Badimon, 2014).

Pannexin-1, Pannexin-2, and Pannexin-3 are hexameric plasma membrane channel-forming proteins, which are structurally similar to Connexins but not gap-junction-forming (Sosinsky et al., 2011). As a member of the Pannexins family, Pannexin-1 channels locates on the cell membrane and releases adenosine triphosphate (ATP), nucleotides and molecules up to 1 kDa into the extracellular space, when activated (Penuela et al., 2013). Activation of Pannexin-1 channels can proceed by various mechanisms, including mechano-stretch, α1-adrenergic/histamine stimulation, oxygen-glucose deprivation and caspase-mediated cleavage of the C-terminal portion, which results in an irreversible opening of the channel pore (Adamson and Leitinger, 2014). A burgeoning body of researches suggests that Pannexin-1 channels contributes to the progression of pathophysiology in many diseases, such as Crohn’s disease, AIDS, melanoma, epilepsy, chronic intestinal inflammation, spinal cord injury, and stroke (Mestas et al., 2005). It is interesting to note that Pannexin-1 channels provides a positive feedback loop for inflammatory responses involved in acute and chronic inflammation-related diseases (Makarenkova and Shestopalov, 2014). Additionally, ATP, ions, glutamate, and second messengers, which are released from injured and necrotic cells through the Pannexin-1 channels, may act as damage-associated molecular pattern (DAMP) signal recognized by TLRs during the inflammatory response. However, none of the previous studies reported the direct role of Pannexin-1 channels in SAH-induced neuroinflammation involves TLRs/NF-κB signaling pathway. Thus, we hypothesized that the Pannexin-1 may participate in inflammatory response after SAH via TLRs/NF-κB -mediated signaling pathway.

## Materials and Methods

### Animals

All experimental protocols including animal research and surgical procedures were approved by the Institutional Animal Care and Use Committee of Nanjing University (Jinling Hospital) and were in accordance with the guidelines for the Care and Use of Laboratory Animals by National Institutes of Health.

Adult male Sprague-Dawley (SD) rats (280–300 g) were purchased from the Animal Center of Jinling Hospital, Nanjing, China. All rats were housed in temperature and humidity controlled animal cages with 12-h light and 12-h dark cycle. All rats were maintained on a standard diet for 1 week before the experimental protocols.

### Rat SAH Model

Briefly, following intraperitoneal anesthesia with 10% chloral hydrate (400 mg/kg), the rat’s head was fixed in the stereotactic frame. Experimental SAH model was produced by stereotaxic insertion of a needle into the prechiasmatic cistern. Non-heparinized fresh autologous arterial blood (0.3 ml) was slowly injected into the prechiasmatic cistern in 20 s period using a syringe pump and aseptic technique according to our previous study (Zhou et al., 2015).

An equal volume of normal saline solution was injected into the prechiasmatic cistern of rats in the sham group. The animals were allowed to recover for 45 min after SAH. After surgery, the rats were returned to their cages and the environmental temperature kept at 23 ± 1°C. Twenty milliliters of 0.9% NaCl solution was injected subcutaneously immediately after the operation to prevent dehydration. Rats died during surgery or surgical recover were excluded, and the procedure was repeated until final group sizes reached the planned experimental number.

### Tissue Preparation

**FIGURE 1:**
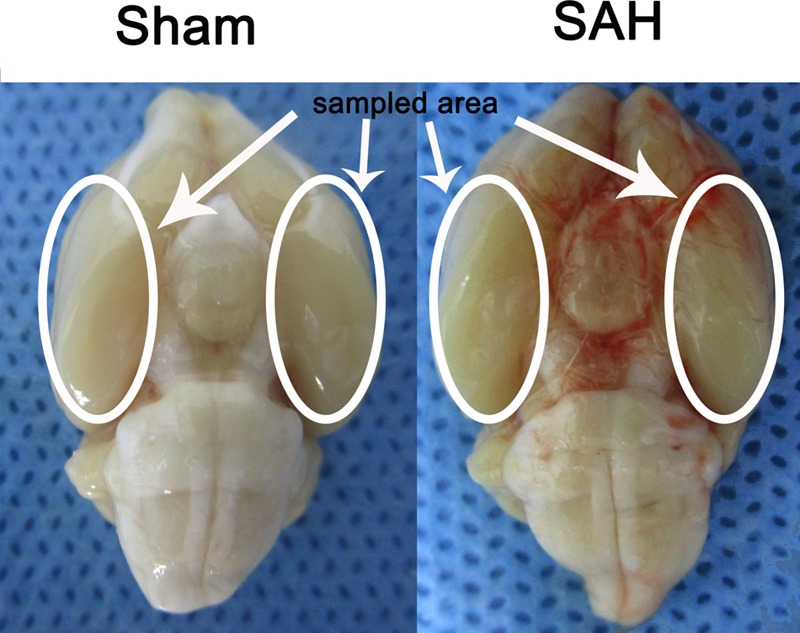
Experimental SAH model in SD rats. Schematic representation of the inferior basal temporal lobe for detection in this study.

In the present study the inferior basal temporal lobe was always stained by blood when the brain was collected. The cortical tissue was separated on ice under a microscope and frozen in liquid nitrogen immediately for molecular biological and biochemical experiments (**Figure 1**).

The remaining rats in each group were anesthetized and intracardially perfused with 0.9% saline followed by 4% paraformaldehyde. After perfusion, the obtained tissue samples were immediately fixed by immersion in 4% paraformaldehyde. The tissues were gradiently dehydrated in 20 and 30% sucrose solution respectively, kept at -80°C, and then sectioned at 6–10 μm for immunofluorescent staining (Li W. et al., 2014).

### Lentiviral Construction and Injection

**FIGURE 2:**
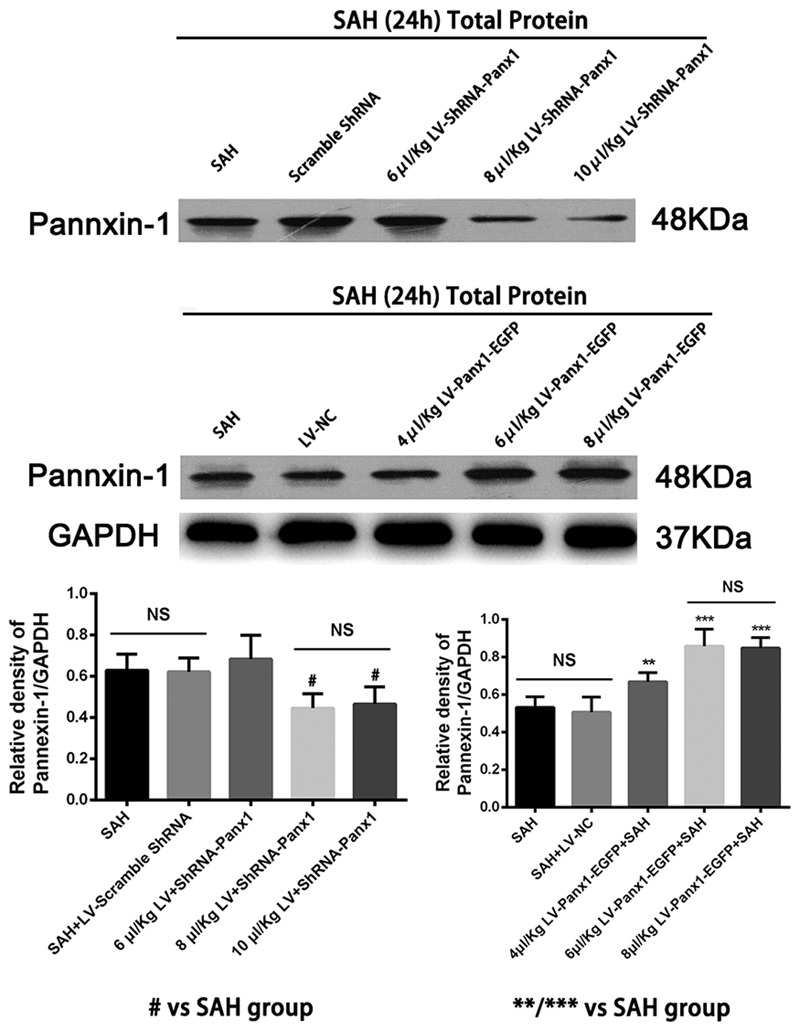
Western blotting showing that LV-ShRNA-Panx1 administration (8 μl/Kg and 10 μl/Kg) markedly depressed the expression levels of Pannexin-1 at 24 h after induction of SAH. No difference was detected between SAH group and SAH + LV-Scramble-ShRNA group. In addition, LV-Panx1-EGFP administration (6 μl/Kg and 8 μl/Kg) dramatically increased the expression levels of Pannexin-1 at the same time point. The experimental SAH model was established at 72 h after lentiviral injection, and we collected samples in each groups at 24 h after SAH. No difference was detected between SAH group and SAH + LV-NC group. GAPDH was used as the loading control. Bars represent the mean ± SD (*n* = 6, each group; ^#^*P* < 0.01 vs. SAH Group; ^∗∗^*P* < 0.01/^∗∗∗^*P* < 0.001 vs. SAH Group; ^NS^*P* > 0.05).

Pannexin-1 channels protein gene knockdown was achieved by transfection of lentiviral vectors expressing Panx1-specific shRNA. The Pannexin-1 shRNA(r) lentiviral particles and a scramble sequence were purchased from Santa Cruz, CA, United States. To establish and maintenance the specific overexpression of Pannexin-1 channels, the Lentiviral vectors LV-Panx1-EGFP-overexpression (NM_199397, Ubi-MCS-3FLAG-CMV-EGFP, GV365) and LV- EGFP were designed, synthesized, and constructed by Genechem, Co., Ltd (Shanghai, China) was generated as a negative control (LV-NC). The viral titer was 1 × 10^8^ TU/mL in LV-Panx1-EGFP and 5 × 10^6^ IFU/ml in LV-ShRNA-Panx1. According to the detection of Lentivirus infection effect, we choose the 8 μl/Kg LV-ShRNA-Panx1 and 6 μl/Kg of LV-Panx1-EGFP were injected into the right lateral ventricles at a rate of 0.2 μl/min with a 10-μL Hamilton microsyringe. The amount of virus in this research was evaluated using the western blot method (**Figure 2**). After completing the infusion, the needle remained in place over a course of 1 min. The experimental SAH model was established at 72 h after lentiviral injection (Zhou et al., 2010).

### Experimental Design

**FIGURE 3:**
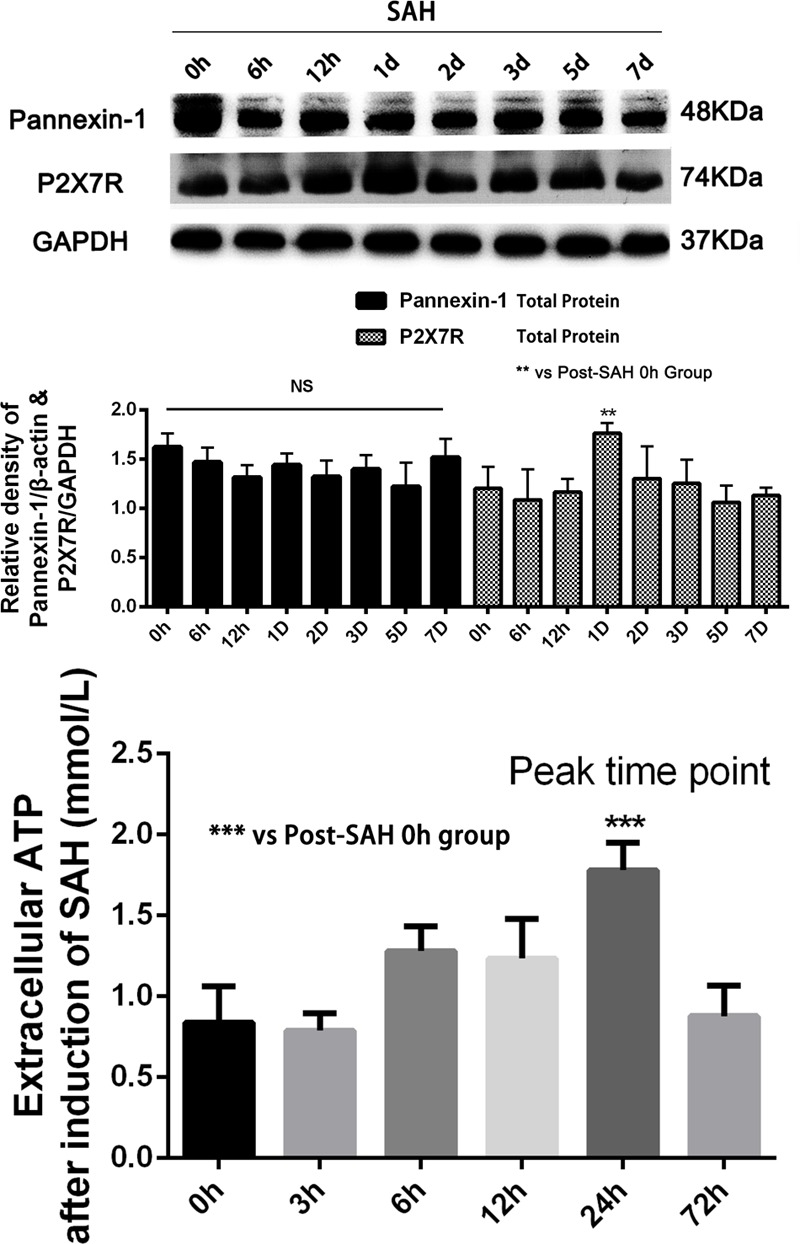
**(Upper)** Western blotting showing Pannexin-1 Channels and P2X7R protein expression in the inferior basal temporal cortex in each time points (0, 6, 12 h, 1, 2, 3, 5, and 7 days) after SAH. There was no statistical difference in expression levels of Pannexin-1 Channels in each time points. Meanwhile, P2X7R (members of the family of ionotropic ATP-gated receptors) protein level was markedly elevated on 24 h after SAH. GAPDH was used as the loading control. Bars represent the mean ± SD (*n* = 6, each group; ^∗∗^*P* < 0.01 vs. Post-SAH 0 h Group; ^NS^*P* > 0.05). **(Bottom)** The levels of extracellular ATP at each time point (0, 3, 6, 12, 24, and 72 h) after induction of SAH. Bars represent the mean ± SD (*n* = 6, each group; ^∗∗∗^*P* < 0.01 vs. Post-SAH 0 h Group; ^NS^*P* > 0.05).

Sprague-Dawley rats were randomly divided into six groups in the experiment: (a) the sham group (*n* = 20); (b) the SAH group (*n* = 20); (c) the SAH + LV-Scramble ShRNA group (*n* = 20); (d) the SAH + LV-ShRNA-Panx1 group (*n* = 20); (e) the SAH + LV-Panx1-EGFP group (*n* = 20); (f) the SAH + LV-NC group (*n* = 20). According to our previous studies of inflammatory response after SAH and peak time of the extracellular ATP concentration and P2X7 receptor (P2X7R) expression (So and Croft, 2013), we collected samples in each groups at 24 h after the establishment of SAH (**Figure 3**). In lentiviral injection groups, the experimental SAH model was established at 72 h after lentiviral or LV-Vehicle (LV-Scramble ShRNA and LV-NC) injection. Respectively, 10 rats in each group were sacrificed for sample collection and tissue assays at 24 h after the SAH model was induced. Meanwhile, the other 10 rats in each group were trained and evaluated in Morris water maze test (MWM).

### Measurement of Extracellular ATP

Extracellular ATP in brain tissues (homogenates without lysis) was quantified with a firefly luciferase based ATP assay kit (Beyotime) according to the manufacturer’s instructions. To avoid ATP catabolism, homogenates was performed with Hanks’ balanced salt solution without calcium and magnesium (Invitrogen Life Technologies, Carlsbad, CA, United States) and 0.1 mM ethylenediaminetetraacetic acid (Sigma-Aldrich). Luminescence produced was measured with a luminometer (Riteau et al., 2010).

### Immunofluorescence Staining

Immunofluorescences (IF) staining was performed according to the immunoreactivity of Pannexin-1 channels in neurons (Anti-NeuN, MAB377X, Alexa Fluor^®^488 conjugated, Millipore, United States, 1:100), astrocytes (Anti-GFAP, 53-9892-80, Alexa Fluor^®^488 conjugated, Affymetrix eBioscience, United States, 1:100), and microglia (Anti-IBA, ab15690, Abcam, United States, 1:500). All protocols of IF were according to our previous study (Li W. et al., 2014). Briefly, frozen temporal lobe sections (4–6 μm) were sliced and blocked with 5% normal fetal bovine serum in PBS containing 0.1% Triton X-100 for 1 h at room temperature prior to incubation with primary antibody overnight at 4°C. After sections were washed three times with PBST for 45 min, they were incubated with proper secondary antibodies (Lot: 121992, Jackson immunoResearch Lab, Inc. 1:100) for 2 h at room temperature. Then the slides were washed with PBST three times for 30 min prior to be counterstained by DAPI for 15 min. After three washes again, the slides were covered by microscopic glass with anti-fade mounting medium for further study. Negative controls were prepared by omitting the primary antibodies. Fluorescence was imaged on a ZEISS Scope.A1 and X-Cite Series 120Q system. The number of positively immunostained cells in each section was counted in 10 microscope fields (at ×400 magnification) throughout the identical regions of the studied brain, and the mean percentage per visual field was calculated.

### Western Blot Analysis

Total/Nuclear Protein Extraction was performed as described previously (Li et al., 2017). The protein concentration was estimated by the Bradford method using the Beyotime Bradford Protein Assay Kit (Beyotime Biotechnology, Shanghai, China). Equal amounts of proteins were separated by 8–15% SDS- polyacrylamide gels (PAGE) and electro-transferred to a polyvinylidene-difluoride (PVDF) membrane (Bio-Rad Lab, Hercules, CA, United States). The membrane was blocked with 5% bovine serum albumin (BSA) for 2 h at room temperature, incubated overnight at 4°C with primary antibodies directed against the TIRAP (Abcam, United States, 1:1000), high-mobility group box 1 (HMGB1, Abcam, United States, 1:5000), TLR4 (Santa Cruz, CA, United States, 1:200), TLR2 (Santa Cruz, CA, United States, 1:200), nuclear factor-κB (NF-κB) P65 (Santa Cruz, CA, United States, 1:200), myeloid differentiation factor 88 (MyD88, Abcam, United States, 1:1000), Pannexin-1 (Abcam, United States, 1:1000), P2X7R (Abcam, United States, 1:1000), Bax (Santa Cruz, CA, United States, 1:200), Bcl-2 (Santa Cruz, CA, United States, 1:200) and Caspase 3 (Bioss, Beijing, China, 1:500), β-actin (diluted 1:5000 in PBST, Bioworld Technology, United States) and GAPDH (diluted 1:1000 in PBST, Bioworld Technology, United States) was used as a loading control. The membrane was washed for 10 min each for six times in TBST, it was incubated in the appropriate HRP-conjugated secondary antibody (Bioworld Technology, United States, 1:1000) for 2 h at room temperature. The blotted protein bands were visualized by enhanced chemiluminescence (ECL) western blot detection reagents (Millipore, United States) and were exposed to Tanon-5200 Chemiluminescent Imaging System. Series of ECL exposures were performed to ensure that non-saturated bands were used for quantification. Relative changes in protein expression were estimated from the mean pixel density using UN-SCAN-IT, normalized to β-actin or GAPDH, and calculated as target protein expression/β-actin or GAPDH expression ratios (Li H. et al., 2014).

### Electrophoretic Mobility Shift Assay (EMSA)

An electrophoretic mobility shift assay (EMSA) commercial kit (Thermo Fisher Scientific, Inc.) assay was used to detect the DNA binding activity of NF-κB according to the manufacturer’s instructions and the methods in our laboratory. The NF-κB oligonucleotide probe (5′-AGTTGAGGGGACTTTCCCAGGC-3′) was synthesized by Biyotime Biotech, Co. Ltd (Shanghai, China) and end-labeled with biotin. The shifted bands were visualized after exposure to film. All protocols of EMSA was performed according to our previous study (Chen et al., 2007).

### Quantitative Real-Time Polymerase Chain Reaction

The mRNA levels of TLR2 and TLR4 were determined by quantitative real-time polymerase chain reaction (PCR). Total cellular RNA was isolated from sample brain using Trizol Reagents (Invitrogen Life Technologies, Carlsbad, CA, United States) as per the manufacturer’s direction. RNA quality was insured by gel visualization and spectrophotometric analysis (OD_260/280_). The primers were synthesized by ShineGene Biotechnology (Shanghai, China) and were shown in **Table 1**. All protocols of QT-PCR were performed according to our previous study (Wang et al., 2013). Test cDNA results were normalized to β-actin. All samples were analyzed in triplicate.

**Table 1:** Polymerase chain reaction (PCR) primer sequences.

Target gene	Sense primer (5′–3′)	Antisense primer (5′–3′)	Size (bp)
TLR2	AAGTAGAAACGGTAACAATACGGAG	AAGAGCAGGGAACCAGAAAGAC	141
TLR4	TATCCAGAGCCGTTGGTGTATCT	AATGAAGATGATGCCAGAGCG	85
β-actin	TGCTATGTTGCCCTAGACTTCG	GTTGGCATAGAGGTCTTTACGG	240

### Enzyme-Linked Immunosorbent Assay (ELISA)

The levels of inflammatory mediators were quantified using specific enzyme-linked immunosorbent assay (ELISA) kits according to the manufacturers’ instructions (TNF-α, IL-1β, IL-6, and IL-18 from Affymetrix eBioscience, Santa Clara, CA, United States) and our previous study (Wang et al., 2012). The inflammatory cytokine contents were calculated as pictogram per milligram protein.

### TUNEL Staining

Apoptosis was determined using terminal deoxynucleotidyl transferase dUTP nick end labeling (TUNEL) and the analysis of the DNA fragmentation assays was based on 3H-thymidine and 5-Bromo-2-deoxy-uridine. All protocols of TUNEL were according to the manufacturer’s protocol (In Situ Cell Death Detection Kit, TMR red from Sigma-Aldrich, St. Louis, MO, United States). Then the slides were washed with PBST three times for 30 min prior to be counterstained by DAPI for 15 min. After three washes, the slides were covered by microscopic glass with anti-fade mounting medium for further fluorescence analysis.

Cell counting was restricted to the temporal cortex. The number of positively stained cells was counted in 10 random microscope fields (at ×400 magnification) in each section were chosen, and the mean percentage per visual field was calculated. A total of six sections from each animal were used for quantification. The final average number of the six sections was regarded as the data for each sample.

### Cognitive and Memory Testing

Spatial learning and memory were assessed by MWM with some modifications including cued learning procedure, spatial acquisition task, reference memory task, and working memory task according to the previous study (Wang et al., 2013). A camera (Chromotrack, San Diego Instruments, San Diego, CA, United States) tracked the movements of rats by videotaping.

The modified MWM for SD rats consisted of a circular water tank filled with thermostatic water (23 ± 2°C) made opaque with Non-toxic black colorant. Suppositional lines divided the maze into four equal-sized quadrants. Behavior testing was performed between 09:00 and 18:00. All animals were housed at a constant temperature of 22 ± 2°C, under a 12-h light/dark cycle, with free access to food and water. Data from the water maze included escape latencies (s) and swimming distance (cm) to find the platform (Szyndler et al., 2006).

### Neurologic Scoring

We used an 18-point scoring system was shown in **Table 2** to evaluate the neurologic function of rats at 24 h after SAH (Sugawara et al., 2008).

**Table 2:** Neurological evaluation after SAH in rats.

Test	Score			
	0	1	2	3
Spontaneous activity (in cage for 5 min)	No movement	Barely moves position	Moves but does not approach at least three sides of cage	Moves and approaches at least three sides of cage
Spontaneous movements of all limbs	No movement	Slight movement of limbs	Moves all limbs but slowly	Move all limbs same as pre-SAH
Movements of forelimbs (outstretching while held by tail)	No outreaching	Slight outreaching	Outreach is limited and less than pre-SAH	Outreach same as pre-SAH
Climbing wall of wire cage		Fails to climb	Climbs weakly	Normal climbing
Reaction to touch on both side of trunk		No response	Weak response	Normal response
Response to vibrissae touch		No response	Weak response	Normal response

### Statistical Analysis

All data were presented as mean ± SD. SPSS 17.0 was used for statistical analysis of the data. All data were subjected to one-way ANOVA. In the semiquantitative analysis of immunofluorescent staining (co-Immunofluorescence and TUNEL), differences between two research groups were determined by the Student’s *t*-test. A value of *P* < 0.05 was considered statistically significant.

## Results

### General Observations and Mortality Rate

There were no statistical changes in physiological parameters [body weight, mean arterial blood pressure (MABP), and body temperature] in any of the experimental groups (data not shown). The mortality rate of rats in the sham group was 0% (0/20 rats), and 22.48% (29/129 rats) in each groups with SAH-surgical procedures. As shown in **Figure 1**, experimental SAH model of rats exhibited blood clots over the basal surface of the brainstem and the area of the circle of Willis.

### Expression and Cellular Localization of Pannexin-1 Channels in the Brain Cortex after Experimental SAH in Rats

To determine the time course of Pannexin-1 channels expression after SAH, rat brain tissues were obtained at different time points after SAH and assayed by Western blot. The cellular localization of Pannexin-1 channels was exhibited by immunofluorescence staining.

**FIGURE 4:**
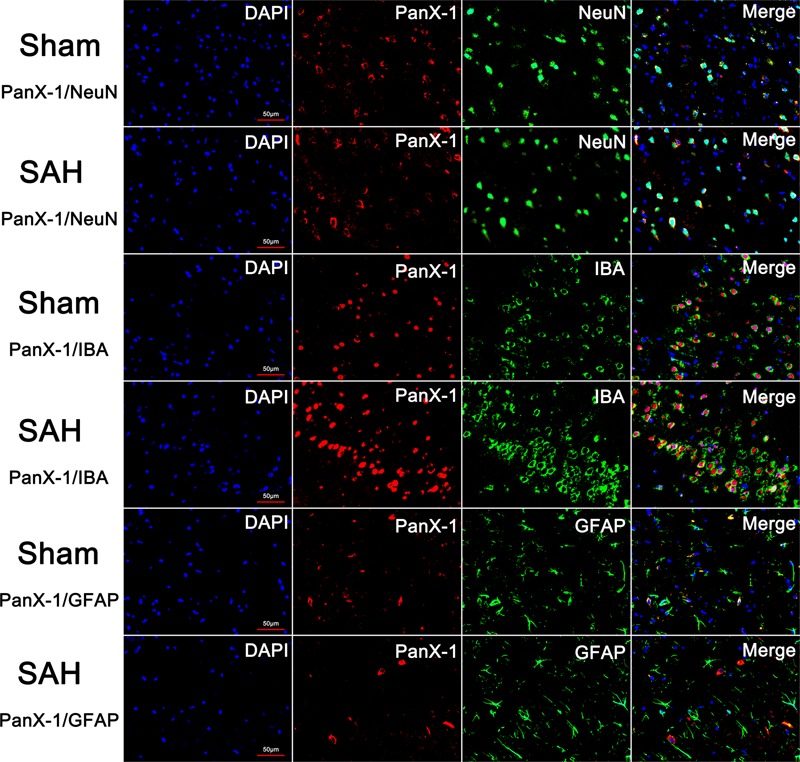
The expression and distribution of Pannexin-1 Channels were detected by double immunofluorescent staining. Pannexin-1 Channels/NeuN, Pannexin-1 Channels/IBA and Pannexin-1 Channels/GFAP co-staining in the Sham group and SAH group (Pannexin-1 Channel = red, NeuN, IBA and GFAP = green, DAPI = blue). According to the result of immunofluorescence staining, at the cellular level, Pannexin-1 Channels were expressed mainly in neurons and microglial cells, whereas infrequently expressed in astrocytes. Data not shown.

There was no statistical difference for the expression of Pannexin-1 channels at each time point and the cellular localization in neurons, astrocytes, and microglia after SAH (**Figures 3, 4**). According to the result of immunofluorescence staining, Pannexin-1 channels were expressed mainly in neurons and in microglial cells, whereas rarely expressed in astrocytes (**Figure 4**).

### Regulation of Pannexin-1 Channels Affected the Expression Levels of TLR2/4/Nf-κB Pathway-Related Agents

**FIGURE 5:**
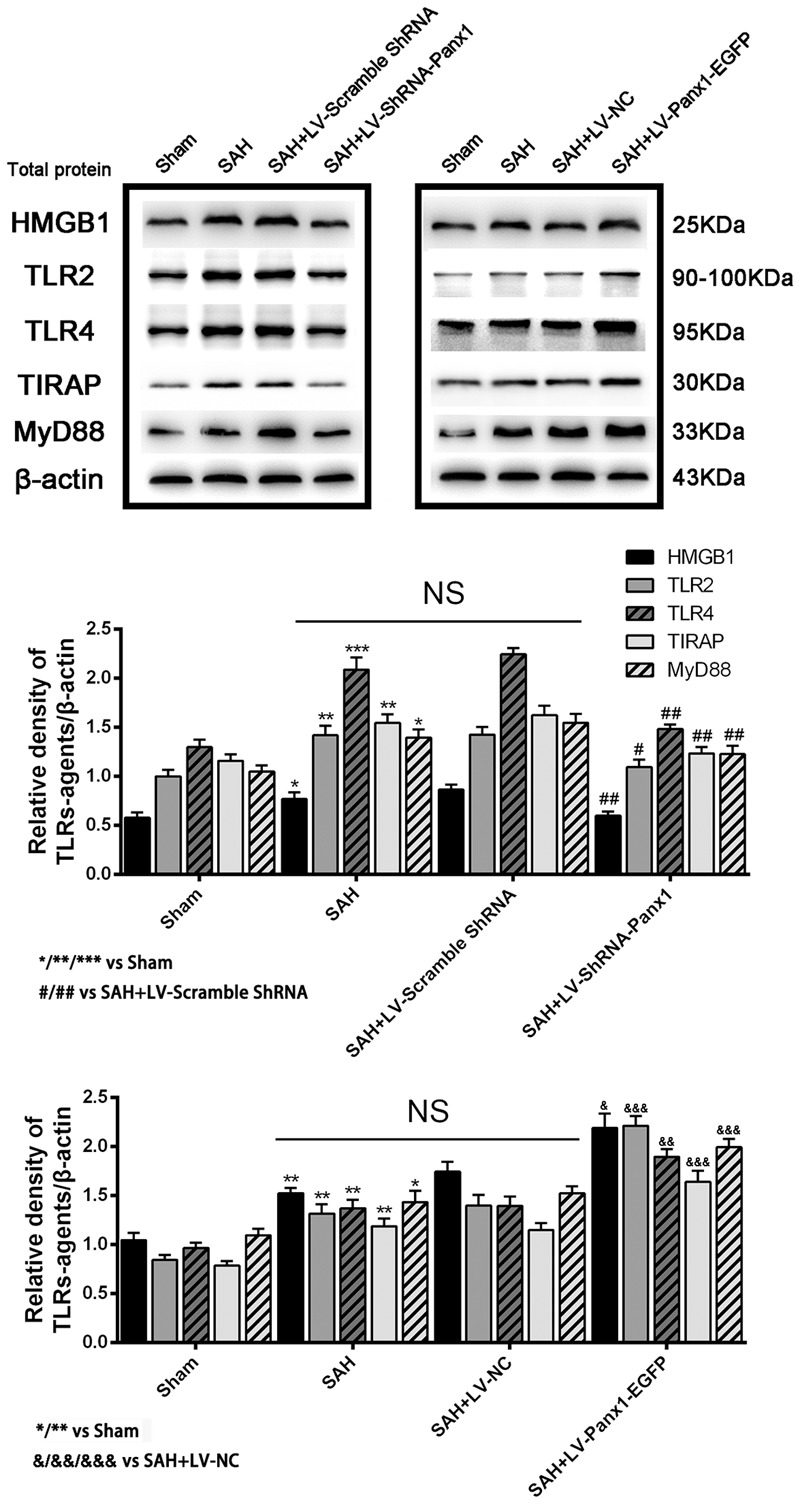
**(Upper)** Representative autoradiogram of HMGB1, TLR2, TLR4, TIRAP, and MyD88 expression in the inferior basal temporal cortex after SAH. The expression of these proteins was elevated in the SAH group and further improved in SAH + LV-Panx1-EGFP group and down-regulated after LV-ShRNA-Panx1 administration. **(Bottom)** Quantitative analysis of the Western blot results shows that these protein levels in SAH groups are significantly higher than Sham group and depressed by LV-ShRNA-Panx1 administration. Otherwise, LV-Panx1-EGFP injection further increased these protein levels as compared with those of SAH + LV-NC group. β-actin was used as the loading control. Bars represent the mean ± SD (*n* = 6, each group; ^∗^*P*/^#^*P*/^&^*P* < 0.05; ^∗∗^*P*/^##^*P*/^&&^*P* < 0.01; ^∗∗∗^*P*/^###^*P*/^&&&^*P* < 0.001; ^NS^*P* > 0.05).

The protein levels of TLR2/4 pathway-related agents were detected by western blot. The levels of TLR2, TLR4, TIRAP, HMGB1, and MyD88 were significantly increased in the cortex in SAH + LV-Panx1-EGFP group as compared with those of SAH + LV-NC group. The expression levels of TLR2, TLR4, TIRAP, HMGB1, and MyD88 in SAH + LV-ShRNA-Panx1 group were significantly lower than those of the SAH + LV-Scramble-ShRNA group (**Figure 5**).

**FIGURE 6:**
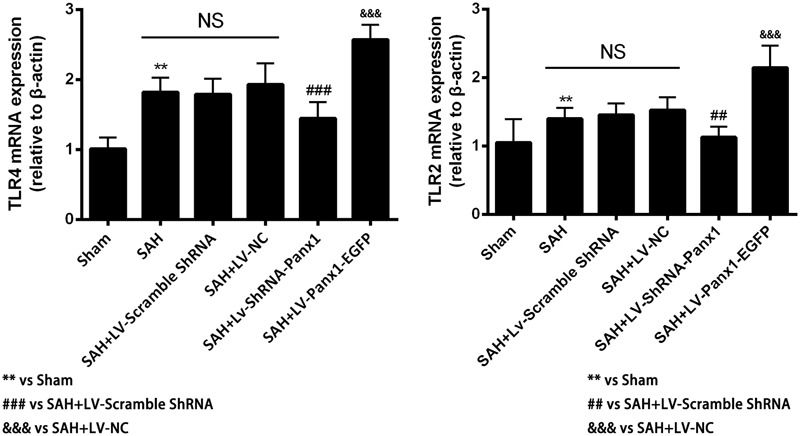
The mRNA expressions of TLR2 and TLR4 were measured by Quantitative real-time PCR in each experimental group. The induction of SAH led to a significantly upward tendency in TLR2 and TLR4 mRNA expressions compared with Sham group. The mRNA expressions of TLR2 and TLR4 were significantly down-regulated with LV-ShRNA-Panx1 administration as compared with SAH + LV-Scramble-ShRNA group and markedly up-regulated with LV-Panx1-EGFP transfection as compared with SAH + LV-NC group. Bars represent the mean ± SD (*n* = 6, each group; ^∗^*P*/^#^*P* < 0.05; ^∗∗^*P*/^##^*P* < 0.01; ^∗∗∗^*P*/^###^*P*/^&&&^*P* < 0.001; ^NS^*P* > 0.05).

The mRNA levels of TLR2/TLR4 were detected by quantitative real-time PCR. The mRNA levels of TLR2 and TLR4 in the SAH + LV-ShRNA-Panx1 group were significantly down-regulated as compared with those of the SAH + LV-Scramble-ShRNA group. Meanwhile, the mRNA expressions of TLR2 and TLR4 in the SAH + LV-Panx1-EGFP group were significantly increased compared with those in SAH + LV-NC group (**Figure 6**).

**FIGURE 7:**
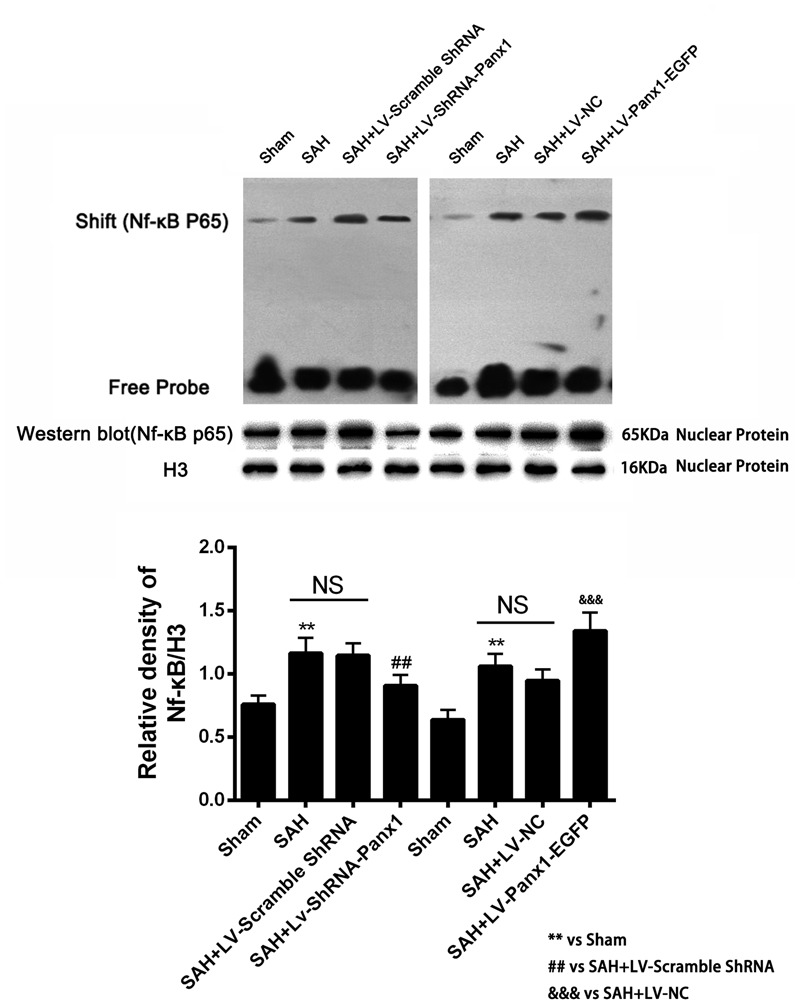
**(Upper)** NF-κB/DNA binding activity measured by EMSA autoradiography was markedly increased after SAH as compared with the Sham group. LV-ShRNA-Panx1 administration significantly suppressed NF-κB activation in SAH + LV-ShRNA-Panx1 group. In contrast, NF-κB activation in SAH + LV-Panx1-EGFP group was higher than SAH + LV-NC group. **(Bottom)** Quantitative analysis of the Western blot results shows that LV-ShRNA-Panx1 administration depressed the nuclear level of NF-κB p65 as compared with the SAH + LV-Scramble-ShRNA group. Furthermore, compared to SAH + LV-NC group, LV-Panx1-EGFP significantly increased the expression level of NF-κB p65 in nuclear protein. H3 was used as the loading control. Bars represent the mean ± SD (*n* = 6, each group; ^∗∗^*P*/^##^*P* < 0.01; ^###^*P*/^&&&^*P* < 0.001; ^NS^*P* > 0.05).

Higher NF-κB binding activity (strong EMSA autoradiography) and expression in nuclear protein was found in the SAH + LV-Panx1-EGFP group compared with SAH + LV-NC group. In SAH + LV-ShRNA-Panx1 group, the NF-κB binding activity was significantly down-regulated along with the same trend in nucleoprotein expression after induction of SAH (**Figure 7**).

### Regulation of Pannexin-1 Channels Altered the Production of Pro-inflammatory Cytokines after Experimental SAH in Rats

**FIGURE 8:**
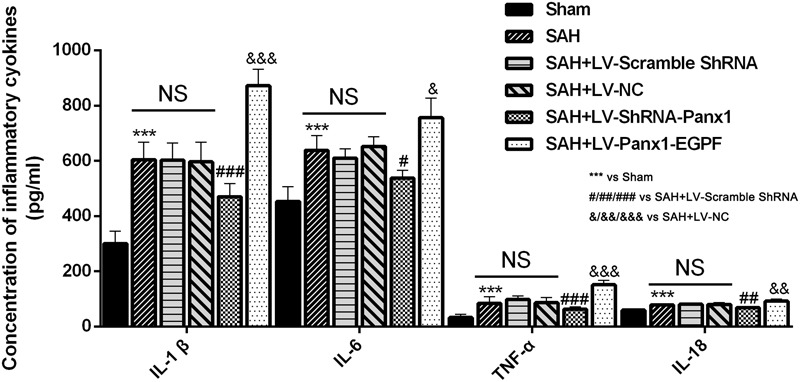
The effects of LV-ShRNA-Panx1 and LV-Panx1-EGFP on levels of IL-1, IL-6, and TNF-α, and IL-18 were measured by ELISA. The concentrations of these inflammatory cytokines were obviously down-regulated by LV-ShRNA-Panx1 administration following SAH. LV-Panx1-EGFP further up-regulated the levels of IL-1β, IL-6, TNF-α, and IL-18 as compared with SAH + LV-NC group. Bars represent the mean ± SD (*n* = 6, each group; ^∗^*P*/^#^*P*/^&^*P* < 0.05; ^∗∗^*P*/^##^*P*/^&&^*P* < 0.01; ^∗∗∗^*P*/^###^*P*/^&&&^*P* < 0.001; ^NS^*P* > 0.05).

Concentrations of IL-1β, TNF-α, IL-6, and IL-18 were lower in the rat brains of SAH + LV-ShRNA-Panx1 group compared with those in the SAH + LV-Scramble-ShRNA group according to the result of ELISA. Cortical levels of the four inflammatory cytokines were significantly elevated after LV-Panx1-EGFP administration compared with those in the SAH + LV-NC group (**Figure 8**).

### Overexpression/Blocking of Pannexin-1 Channels Promoted/Depressed Cell Apoptosis after SAH in Rats

The expression of the apoptosis-related protein (Bcl-2, Bax, and cleaved Caspase-3 were assessed by western blot) and TUNEL staining were performed to demonstrate the role of Pannexin-1 channels in neuronal cells apoptosis.

**FIGURE 9:**
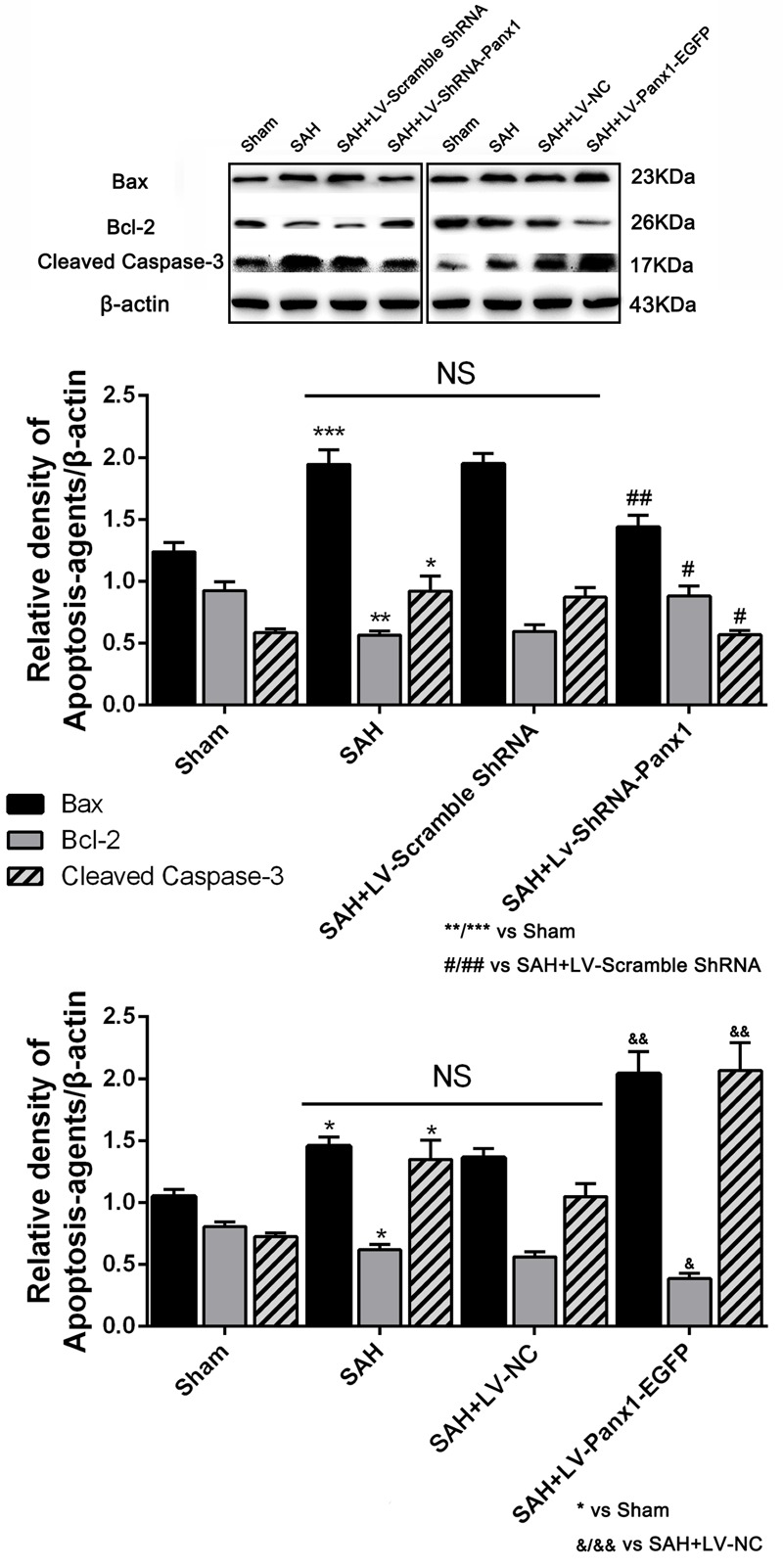
The expression levels of apoptosis associated proteins were assessed by Western blot analysis.Quantitative analysis of representative autoradiogram of Bax, Bcl-2, and cleaved Caspase-3 shows that LV-ShRNA-Panx1 administration significantly suppressed the expression of apoptosis associated proteins as compared with SAH + LV-Scramble-ShRNA group. Likewise, in LV-Panx1-EGFP group, Bax and cleaved Caspase-3 protein levels were markedly higher than that in SAH + LV-NC group. β-actin was used as the loading control. Bars represent the mean ± SD (*n* = 6, each group; ^∗^*P*/^#^*P*/^&^*P* < 0.05; ^∗∗^*P*/^##^*P*/^&&^*P* < 0.01; ^∗∗∗^*P* < 0.001; ^NS^*P* > 0.05).

**FIGURE 10:**
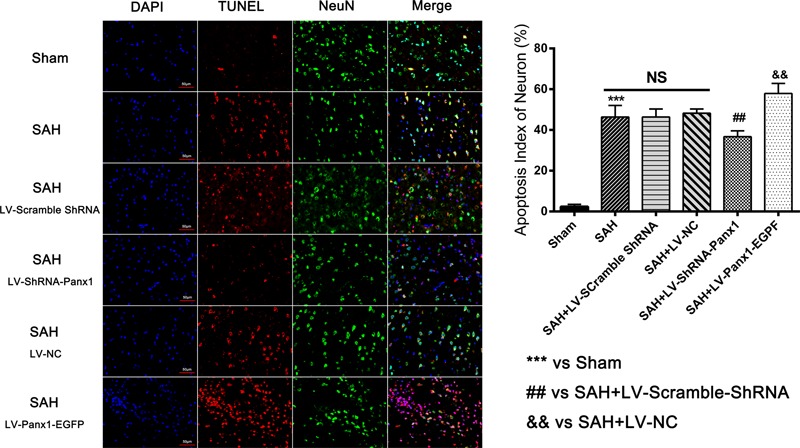
Co-immunofluorescent staining shows that TUNEL-positive cells co-localized with the neurons marker (NeuN) in the overlapped images. It showed that the number of TUNEL-positive neurons was higher in SAH group as compared to that in Sham group. In LV-Panx1-EGFP group, the number of TUNEL-positive neurons was even much higher than SAH + LV-NC group. LV-ShRNA-Panx1 attenuates the level of TUNEL-positive neurons as compared with SAH + LV-Scramble-ShRNA group (TUNEL = red, NeuN = green, DAPI = blue, Scale bars 20 μm). Bars represent the mean ± SD (*n* = 6, each group; ^##^*P*/^&&^*P* < 0.01; ^∗∗∗^*P* < 0.001; ^NS^*P* > 0.05).

The levels of Bax and cleaved Caspase-3 were down-regulated after the LV-ShRNA-Panx1 injection compared with those in the SAH + LV-Scramble-ShRNA groups; In addition, the level of Bcl-2 was up-regulated as compared with the SAH + LV-NC group, administration of LV-Panx1-EGFP up-regulated the levels of Bax and cleaved Caspase-3, while down-regulated Bcl-2 expression (**Figure 9**). The percentage of positive TUNEL-neurons in the brain was decreased by LV-ShRNA-Panx1, and elevated by LV-Panx1-EGFP after SAH (**Figure 10**).

### Modulation Effects of Pannexin-1 Channels on Neurological Function

**FIGURE 11:**
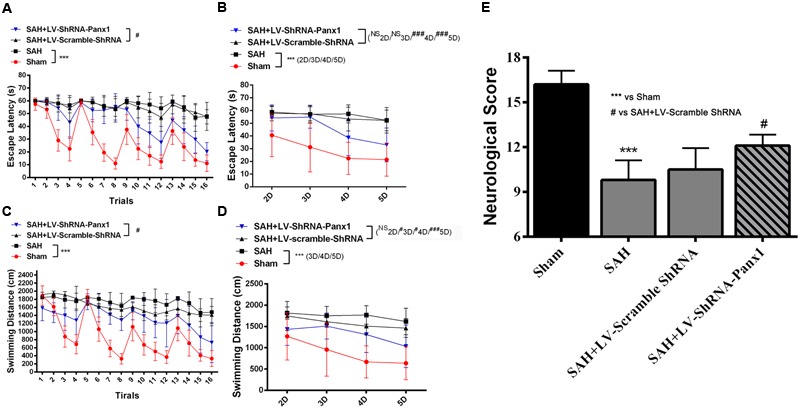
Spatial learning/memory was evaluated in the Morris Water Maze (MWM). Escape latency (s) and traveled distance (cm) over total 16 trials **(A,C)** and averaged for each group and each day **(B,D)** over days 2–5. The SAH group showed markedly longer escape latency and traveled distance (**A,C**, *n* = 10, ^∗∗∗^*P* < 0.001 repeated ANOVA) over the 16 trials as compared to Sham groups. The SAH + LV-ShRNA-Panx1 group exhibited obviously shorter escape latency and traveled distance (**A,C**, *n* = 10, ^#^*P* < 0.05 repeated ANOVA) over the 16 trials than the SAH + LV-Scramble-ShRNA group. The averaged data showed a similar significantly increased escape latency and traveled distance (**B,D**, *n* = 10, ^∗∗∗^*P* < 0.001 one-way ANOVA) in SAH group on Day 2, Day 3, Day 4, and Day 5 as compared to Sham group. In the SAH + LV-ShRNA-Panx1 group, the averaged data showed significantly shorter escape latency (**B**, *n* = 10, ^###^*P* < 0.001 one-way ANOVA) on Day 4 and Day 5 and traveled distance (**D**, *n* = 10, ^#^*P* < 0.05; ^###^*P* < 0.001 one-way ANOVA) on Day 3, Day 4, and Day 5 than the SAH + LV-Scramble-ShRNA group. LV-ShRNA-PANX1 treatment elevated the neurological scores compared with the SAH + LV-Scramble-ShRNA group at 24 h after SAH (**E**, *n* = 10, ^∗∗∗^*P* < 0.001, ^#^*P* < 0.05).

In water Morris Maze, all experimental rats were arranged to process four trials per days from Day 2 to Day 5 (**Figures 11A–D**). Repeated measures ANOVA indicated a significant difference in escape latency (**Figure 11A**, ^∗∗∗^*P* < 0.001 repeated ANOVA) and in traveled distance (**Figure 11C**, ^∗∗∗^*P* < 0.001 repeated ANOVA) between the SAH group and Sham group. The SAH + LV-ShRNA-Panx1 group exhibited significantly shorter escape latency and traveled distance compared with the SAH + LV-Scramble-ShRNA group (**Figures 11A,C**, ^#^*P* < 0.05 repeated ANOVA).

The averaged data of each group per day showed significantly increased escape latency (**Figure 11B**, ^∗∗∗^*P* < 0.001 one-way ANOVA) and longer traveled distance (**Figure 11D**, ^∗∗∗^*P* < 0.001 one-way ANOVA) in SAH group as compared to Sham group. In the SAH + LV-ShRNA-Panx1 group, the averaged data showed significantly shorter escape latency and traveled distance (**Figures 11B,D**, ^NS^*P* > 0.05, ^#^*P* < 0.05, ^##^*P* < 0.01, ^###^*P* < 0.001 one-way ANOVA) compared with the SAH + LV-Scramble-ShRNA group.

For intuitionistic understanding of the symptoms and pathologic development following SAH, neurological scores were also evaluated. The neurological scores were improved after the administration of LV-ShRNA-PANX1 compared with the SAH + LV-Scramble-ShRNA group (**Figure 11E**).

## Discussion

In the present study, for the first time, we demonstrated that Pannexin-1 channels were involved in TLR2/TLR4/NF-κB-mediated inflammatory responses following SAH. Depression of Pannexin-1 channels with LV-ShRNA-Panx1, down-regulated the agents related to TLR2/TLR4/NF-κB signaling pathway, palliated neural apoptosis and ameliorated SAH-induced neurological dysfunction. In addition, elevated expression of Pannexin-1 channels further exacerbated the inflammatory brain injury linked to TLR2/TLR4/NF-κB signaling pathway. These results support the notion that Pannexin-1 channels are involved in the SAH-induced cerebral inflammatory response and facilitate the development of EBI following rats SAH model.

Increasing evidence has demonstrated the critical role of EBI in the development and maintenance of secondary brain injury after SAH. The cascade of events that occur within the first 72 h after SAH are responsible for the initial signs and symptoms of patients with SAH, which is believed to be a precursor for both delayed vasospasm and DINDs (Chen et al., 2014). In addition, a range of pathological processes that occurs early can trigger a number of devastating cascades that lead to BBB dysfunction, inflammation, necrosis, apoptosis, and oxidative stress (Kanamada, 2010). Additionally, there is no doubt that inflammation could contribute to EBI and lead to poor outcomes (Miller et al., 2014). As an extremely important part of innate immunity, toll-like receptors (TLRs) are host-cell surface receptors that detect the pathogen-associated molecular patterns (PAMPs) and DAMPSs that correspond to endogenous ligands released after tissue injury or cellular stress, such as ATP, histones, heat-shock proteins, mRNA, high-mobility group box-1 protein (HMGB1), and mitochondrial proteins (Aguirre et al., 2013; Fadakar et al., 2014). During the inflammatory response, activation of TLRs triggers the activation of several transcription factors, such as NF-κB, activator protein-1, and IFN-regulatory factor-3/-7, leading to the upregulation of various cytokines and chemokines at the early stage of SAH (Aguirre et al., 2013).

Previous studies verified that, in particular, Pannexin-1 channels promotes activation of the inflammasome in macrophages, neurons and microglia, regulates chemotaxis of phagocytes during apoptosis, promotes T-cell activation, induces neuronal death in enteric colitis, and mediates ‘find-me’ signal release and membrane permeability during apoptosis (Chekeni et al., 2010; Lohman et al., 2015). Excess of extracellular ATP is an activator of both innate and acquired immunities, acting as a chemotactic factor for neutrophils and a strong regulator of activation, death, and survival of microglia (Zhang et al., 2010; Harada et al., 2011; Junger, 2011). As membrane receptors, it is possible that Pannexin-1 channels and TLR2/TLR4 may generate a “membrane to membrane” effect, which facilitates ATP and other molecules released from Pannexin-1 channels to be recognized as DAMPs or PAMPs by TLRs, thus activating the cascade of TLRs/NF-κB-mediated inflammatory response. Our results demonstrated that Pannexin-1 channels could regulate the TLR2/TLR4/NF-κB pathway, providing a framework for understanding the link between the Pannexin-1 channels and TLRs.

In addition, extracellular ATP serves as a danger signal by binding to P2X7R and then activating NALP3 and Caspase-1, which leads to the maturation and release of IL-1β, and the eventual formation of the NALP3 inflammasome (Bours et al., 2006; Mariathasan et al., 2006; Sutterwala et al., 2006), as well as the release of HMGB1 (Toki et al., 2015). Furthermore, P2X7R could be activated by ATP released from pre-synaptic neurons, which in turn phosphorylates Pannexin-1 channels by Src-famliy kinases (Bravo et al., 2015). Recently Pannexin-1 channels was also demonstrated to form the large pore of the P2X7R (Pelegrin and Surprenant, 2006; Locovei et al., 2007). P2X7R plays a critical role in stress-induced inflammatory responses and its activation by extracellular ATP from Pannexin-1 channels results in release of interleukins from macrophages (Pelegrin and Surprenant, 2006). Besides, Pannexin-1 associates with the inflammasome that links the P2X7 receptor resulting in activation and eventual release of cytokines (Silverman et al., 2009). Here, extracellular ATP acts as a bridge connecting P2X7R/Pannexin-1 and TLRs, which is consistent with the observation of ATP-induced activation of TLRs. The activation of multiple TLRs, coupled with elevated ATP levels and subsequent Pannexin-1/P2X7R activation may represent an important route in inflammatory response following SAH.

Numerous clinical studies showed that approximately 50% of SAH survivors have cognitive or neurobehavioral dysfunction. The mechanisms involved in cognitive or neurobehavioral deficits attribute to the instant brain injury or may be a consequence of cerebrovascular dysfunction and DINDs (Jeon et al., 2009). According to our findings, blockade of Pannexin-1 channels alleviates neurological function deficits in this rats SAH model accompanied with the depression of TLR2/TLR4/NF-κB pathway-related agents. In addition, our results also showed that Pannexin-1 channels could regulate the expression levels of apoptosis-related proteins (Bcl-2, Bax, and cleaved Caspase-3) and the proportion of positive Neuro-TUNEL cells, and thereby intervene in the neuronal apoptosis after SAH. Therefore, it is plausible to believe that Pannexin-1 channels holds great potential in improving cognitive or neurobehavioral function in the early stage of SAH due to its anti-inflammatory and anti-apoptotic effects.

To the best of our knowledge, this is the first report to demonstrate the implication of Pannexin-1 channels in TLR2/TLR4/Nf-κB mediated inflammatory responses after SAH. However, several limitations of our study should be pointed out. First, LV-ShRNA-Panx1 may not act as a highly efficient inhibitor of pannexin-1. Further, future studies need to confirm our investigations associated with generation of gene knock-out mice successfully. Second, according to our previous studies on inflammatory response after SAH and peak time of extracellular ATP, we chose only one time point (24 h after SAH) in our study, the role of Pannexin-1 channels in TLRs/NF-κB mediated signaling pathway at diffident time points after SAH is still needed to be investigated in our future studies. In addition, given the current research is a pilot study, further studies are still needed to validate the exact role of Pannexin-1 channels in SAH and other cerebrovascular diseases.

## Conclusion

Pannexin-1 channels contributes to EBI and cognitive/neurobehavioral deficits via TLR2/TLR4/Nf-κB mediated inflammatory signaling pathway after SAH, suggesting the potential role of Pannexin-1 as a therapeutic target in improving the outcome of SAH patients. Thus, more mechanistic studies are needed to provide deep insight into molecular mechanisms of Pannexin-1 channels in SAH.

## Author Contributions

L-YW designed the study, performed the SAH model and biochemical analysis and wrote the manuscript. Z-NY. performed the Quantitative real-time polymerase chain reaction assay, electrophoretic mobility shift assay, and enzyme-linked immunosorbent assay. C-HZ, C-XW, and G-BX performed the immunofluorescence staining and the animal studies. X-SZ, Y-YG, and Z-HZ contributed to the Western blotting. ZZ, M-LZ, and J-PL designed the animal studies and measurement of ATP. C-HH and J-XS contributed to the design and analysis of the study and wrote the manuscript. All authors approved the final version of the manuscript.

## Conflict of Interest Statement

The authors declare that the research was conducted in the absence of any commercial or financial relationships that could be construed as a potential conflict of interest.

## References

[B1] Adamson S. E. Leitinger N. (2014). The role of pannexin1 in the induction and resolution of inflammation. *FEBS Lett.* 588 1416–1422. 10.1016/j.febslet.2014.03.009 24642372 PMC4060616

[B2] Aguirre A. Maturana C. J. Harcha P. A. Saez J. C. (2013). Possible involvement of TLRs and hemichannels in stress-induced CNS dysfunction via mastocytes, and glia activation. *Mediators Inflamm.* 2013:893521 10.1155/2013/893521 PMC3713603 23935250

[B3] Ascenzi P. Bocedi A. Visca P. Altruda F. Tolosano E. Beringhelli T. (2005). Hemoglobin and heme scavenging. *IUBMB Life* 57 749–759. 10.1080/15216540500380871 16511968

[B4] Bours M. Swennen E. Di Virgilio F. Cronstein B. Dagnelie P. (2006). Adenosine 5’-triphosphate and adenosine as endogenous signaling molecules in immunity and inflammation. *Pharmacol. Ther.* 112 358–404. 10.1016/j.pharmthera.2005.04.013 16784779

[B5] Bravo D. Maturana C. J. Pelissier T. Hernandez A. Constandil L. (2015). Interactions of pannexin 1 with NMDA and P2X7 receptors in central nervous system pathologies: Possible role on chronic pain. *Pharmacol. Res.* 101 86–93. 10.1016/j.phrs.2015.07.016 26211949

[B6] Chekeni F. B. Elliott M. R. Sandilos J. K. Walk S. F. Kinchen J. M. Lazarowski E. R. (2010). Pannexin 1 channels mediate ‘find-me’ signal release and membrane permeability during apoptosis. *Nature* 467 863–867. 10.1038/nature09413 20944749 PMC3006164

[B7] Chen G. Shi J. X. Hang C. H. Xie W. Liu J. Liu X. (2007). Inhibitory effect on cerebral inflammatory agents that accompany traumatic brain injury in a rat model: a potential neuroprotective mechanism of recombinant human erythropoietin (rhEPO). *Neurosci. Lett.* 425 177–182. 10.1016/j.neulet.2007.08.022 17825990

[B8] Chen S. Feng H. Sherchan P. Klebe D. Zhao G. Sun X. (2014). Controversies and evolving new mechanisms in subarachnoid hemorrhage. *Prog. Neurobiol.* 115 64–91. 10.1016/j.pneurobio.2013.09.002 24076160 PMC3961493

[B9] Chen Y. Li Q. Tang J. Feng H. Zhang J. H. (2015). The evolving roles of pericyte in early brain injury after subarachnoid hemorrhage. *Brain Res.* 1623 110–122. 10.1016/j.brainres.2015.05.004 25982598 PMC4569518

[B10] Connolly E. S. Jr. Rabinstein A. A. Carhuapoma J. R. Derdeyn C. P. Dion J. Higashida R. T. (2012). Guidelines for the management of aneurysmal subarachnoid hemorrhage: a guideline for healthcare professionals from the American Heart Association/american Stroke Association. *Stroke* 43 1711–1737. 10.1161/STR.0b013e3182587839 22556195

[B11] Cossu G. Messerer M. Oddo M. Daniel R. T. (2014). To look beyond vasospasm in aneurysmal subarachnoid haemorrhage. *Biomed Res. Int.* 2014:628597 10.1155/2014/628597 PMC4055362 24967389

[B12] Fadakar K. Dadkhahfar S. Esmaeili A. Rezaei N. (2014). The role of Toll-like receptors (TLRs) in stroke. *Rev. Neurosci.* 25 699–712. 10.1515/revneuro-2013-0069 24807166

[B13] Fujii M. Yan J. Rolland W. B. Soejima Y. Caner B. Zhang J. H. (2013). Early brain injury, an evolving frontier in subarachnoid hemorrhage research. *Transl. Stroke Res.* 4 432–446. 10.1007/s12975-013-0257-2 23894255 PMC3719879

[B14] Harada K. Hide I. Seki T. Tanaka S. Nakata Y. Sakai N. (2011). Extracellular ATP differentially modulates Toll-like receptor 4-mediated cell survival and death of microglia. *J. Neurochem.* 116 1138–1147. 10.1111/j.1471-4159.2011.07170.x 21210814

[B15] Jeon H. Ai J. Sabri M. Tariq A. Shang X. Chen G. (2009). Neurological and neurobehavioral assessment of experimental subarachnoid hemorrhage. *BMC Neurosci.* 10:103 10.1186/1471-2202-10-103 PMC2749856 19706182

[B16] Junger W. G. (2011). Immune cell regulation by autocrine purinergic signalling. *Nat. Rev. Immunol.* 11 201–212. 10.1038/nri2938 21331080 PMC4209705

[B17] Kanamada A. S. (2010). Connecting the early brain injury of aneurysmal subarachnoid hemorrhage to clinical practice. *Turk. Neurosurg.* 20 159–166. 10.5137/1019-5149.JTN.2714-09.0 20401843

[B18] Li H. Wu W. Sun Q. Liu M. Li W. Zhang X. S. (2014). Expression and cell distribution of receptor for advanced glycation end-products in the rat cortex following experimental subarachnoid hemorrhage. *Brain Res.* 1543 315–323. 10.1016/j.brainres.2013.11.023 24291745

[B19] Li H. Yu J. S. Zhang D. D. Yang Y. Q. Huang L. T. Yu Z. (2017). Inhibition of the receptor for advanced glycation end-products (RAGE) attenuates neuroinflammation while sensitizing cortical neurons towards death in experimental subarachnoid hemorrhage. *Mol. Neurobiol.* 54 755–767. 10.1007/s12035-016-9703-y 26768594

[B20] Li W. Ling H. P. You W. C. Liu H. D. Sun Q. Zhou M. L. (2014). Elevated cerebral cortical CD24 levels in patients and mice with traumatic brain injury: a potential negative role in nuclear factor kappab/inflammatory factor pathway. *Mol. Neurobiol.* 49 187–198. 10.1007/s12035-013-8509-4 23881416

[B21] Locovei S. Scemes E. Qiu F. Spray D. C. Dahl G. (2007). Pannexin1 is part of the pore forming unit of the P2X 7 receptor death complex. *FEBS Lett.* 581 483–488. 10.1016/j.febslet.2006.12.056 17240370 PMC1868681

[B22] Lohman A. W. Leskov I. L. Butcher J. T. Johnstone S. R. Stokes T. A. Begandt D. (2015). Pannexin 1 channels regulate leukocyte emigration through the venous endothelium during acute inflammation. *Nat. Commun.* 6:7965 10.1038/ncomms8965 PMC4824045 26242575

[B23] Lucke-Wold B. P. Logsdon A. F. Manoranjan B. Turner R. C. McConnell E. Vates G. E. (2016). Aneurysmal subarachnoid hemorrhage and neuroinflammation: a comprehensive review. *Int. J. Mol. Sci.* 17:497 10.3390/ijms17040497 PMC4848953 27049383

[B24] Makarenkova H. P. Shestopalov V. I. (2014). The role of pannexin hemichannels in inflammation and regeneration. *Front. Physiol.* 5:63 10.3389/fphys.2014.00063 PMC3933922 24616702

[B25] Mariathasan S. Weiss D. S. Newton K. McBride J. O’Rourke K. Roose-Girma M. (2006). Cryopyrin activates the inflammasome in response to toxins and ATP. *Nature* 440 228–232. 10.1038/nature04515 16407890

[B26] Mestas J. Crampton S. P. Hori T. Hughes C. C. (2005). Endothelial cell co-stimulation through OX40 augments and prolongs T cell cytokine synthesis by stabilization of cytokine mRNA. *Int. Immunol.* 17 737–747. 10.1093/intimm/dxh255 15908450

[B27] Miller B. A. Turan N. Chau M. Pradilla G. (2014). Inflammation, vasospasm, and brain injury after subarachnoid hemorrhage. *Biomed. Res. Int.* 2014:384342 10.1155/2014/384342 PMC4106062 25105123

[B28] Pelegrin P. Surprenant A. (2006). Pannexin-1 mediates large pore formation and interleukin-1β release by the ATP-gated P2X7 receptor. *EMBO J.* 25 5071–5082. 10.1038/sj.emboj.7601378 17036048 PMC1630421

[B29] Penuela S. Gehi R. Laird D. W. (2013). The biochemistry and function of pannexin channels. *Biochim. Biophys. Acta* 1828 15–22. 10.1016/j.bbamem.2012.01.017 22305965

[B30] Pradilla G. Chaichana K. L. Hoang S. Huang J. Tamargo R. J. (2010). Inflammation and cerebral vasospasm after subarachnoid hemorrhage. *Neurosurg. Clin. N. Am.* 21 365–379. 10.1016/j.nec.2009.10.008 20380976

[B31] Riteau N. Gasse P. Fauconnier L. Gombault A. Couegnat M. Fick L. (2010). Extracellular ATP is a danger signal activating P2X7 receptor in lung inflammation and fibrosis. *Am. J. Respir. Crit. Care Med.* 182 774–783. 10.1164/rccm.201003-0359OC 20522787

[B32] Silverman W. R. de Rivero Vaccari J. P. Locovei S. Qiu F. Carlsson S. K. Scemes E. (2009). The pannexin 1 channel activates the inflammasome in neurons and astrocytes. *J. Biol. Chem.* 284 18143–18151. 10.1074/jbc.M109.004804 19416975 PMC2709345

[B33] So T. Croft M. (2013). Regulation of PI-3-kinase and Akt signaling in T lymphocytes and other cells by TNFR family molecules. *Front. Immunol.* 4:139 10.3389/fimmu.2013.00139 PMC3675380 23760533

[B34] Sosinsky G. E. Boassa D. Dermietzel R. Duffy H. S. Laird D. W. MacVicar B. (2011). Pannexin channels are not gap junction hemichannels. *Channels* 5 193–197. 10.4161/chan.5.3.15765 21532340 PMC3704572

[B35] Sugawara T. Ayer R. Jadhav V. Zhang J. H. (2008). A new grading system evaluating bleeding scale in filament perforation subarachnoid hemorrhage rat model. *J. Neurosci. Methods* 167 327–334. 10.1016/j.jneumeth.2007.08.004 17870179 PMC2259391

[B36] Sutterwala F. S. Ogura Y. Szczepanik M. Lara-Tejero M. Lichtenberger G. S. Grant E. P. (2006). Critical role for NALP3/CIAS1/Cryopyrin in innate and adaptive immunity through its regulation of caspase-1. *Immunity* 24 317–327. 10.1016/j.immuni.2006.02.004 16546100

[B37] Szyndler J. Piechal A. Blecharz-Klin K. Skórzewska A. Maciejak P. Walkowiak J. (2006). Effect of kindled seizures on rat behavior in water Morris maze test and amino acid concentrations in brain structures. *Pharmacol. Rep.* 58 75–82. 16531633

[B38] Toki Y. Takenouchi T. Harada H. Tanuma S. Kitani H. Kojima S. (2015). Extracellular ATP induces P2X7 receptor activation in mouse Kupffer cells, leading to release of IL-1beta, HMGB1, and PGE2, decreased MHC class I expression and necrotic cell death. *Biochem. Biophys. Res. Commun.* 458 771–776. 10.1016/j.bbrc.2015.02.011 25681768

[B39] van Beijnum J. R. Buurman W. A. Griffioen A. W. (2008). Convergence and amplification of toll-like receptor (TLR) and receptor for advanced glycation end products (RAGE) signaling pathways via high mobility group B1 (HMGB1). *Angiogenesis* 11 91–99. 10.1007/s10456-008-9093-5 18264787

[B40] Vergouwen M. D. Ilodigwe D. Macdonald R. L. (2011). Cerebral infarction after subarachnoid hemorrhage contributes to poor outcome by vasospasm-dependent and-independent effects. *Stroke* 42 924–929. 10.1161/STROKEAHA.110.597914 21311062

[B41] Vilahur G. Badimon L. (2014). Ischemia/reperfusion activates myocardial innate immune response: the key role of the toll-like receptor. *Front. Physiol.* 5:496 10.3389/fphys.2014.00496 PMC4270170 25566092

[B42] Wang Z. Ma C. Meng C. J. Zhu G. Q. Sun X. B. Huo L. (2012). Melatonin activates the Nrf2-ARE pathway when it protects against early brain injury in a subarachnoid hemorrhage model. *J. Pineal Res.* 53 129–137. 10.1111/j.1600-079X.2012.00978.x 22304528

[B43] Wang Z. Wu L. You W. Ji C. Chen G. (2013). Melatonin alleviates secondary brain damage and neurobehavioral dysfunction after experimental subarachnoid hemorrhage: possible involvement of TLR4-mediated inflammatory pathway. *J. Pineal Res.* 55 399–408. 10.1111/jpi.12087 24007200

[B44] Zhang Q. Raoof M. Chen Y. Sumi Y. Sursal T. Junger W. (2010). Circulating mitochondrial DAMPs cause inflammatory responses to injury. *Nature* 464 104–107. 10.1038/nature08780 20203610 PMC2843437

[B45] Zhou C. Xie G. Wang C. Zhang Z. Chen Q. Zhang L. (2015). Decreased progranulin levels in patients and rats with subarachnoid hemorrhage: a potential role in inhibiting inflammation by suppressing neutrophil recruitment. *J. Neuroinflammation* 12 200 10.1186/s12974-015-0415-4 PMC4630923 26527034

[B46] Zhou L. Li F. Xu H.-B. Luo C.-X. Wu H.-Y. Zhu M.-M. (2010). Treatment of cerebral ischemia by disrupting ischemia-induced interaction of nNOS with PSD-95. *Nat. Med.* 16 1439–1443. 10.1038/nm.2245 21102461

